# Zinc and animal health: an in-depth exploration of its role in physiological functions and regulatory molecular mechanisms

**DOI:** 10.1186/s40104-025-01301-x

**Published:** 2025-12-09

**Authors:** Zhaolong Cai, Jingjing Wang, Yuxi Zhang, Xiaohan Li, Jilong Luo, Xuejiao Gao, Mengyao Guo

**Affiliations:** College of Veterinary Medicine, Northeast Agricultural University, Harbin, 150030 People’s Republic of China

**Keywords:** Animal health, Growth and development, Immune function, Reproductive performance, Zinc deficiency and excess

## Abstract

Zinc, an essential trace element, plays a pivotal role in maintaining animal health and physiological functions. This review comprehensively examines zinc metabolism—including absorption dynamics across species (poultry, ruminants, and non-ruminants), transport mechanisms, storage in tissues, e.g., the liver, and excretion pathways—and its multifaceted effects on animal health. Zinc critically regulates aspects of growth and development, particularly bone formation, as its deficiency induces skeletal deformities in young animals. It modulates immune function through zinc finger proteins, influencing immune organ integrity, lymphocyte proliferation, and cytokine expression. Reproductive performance is significantly affected by zinc, with its deficiency causing impaired spermatogenesis; delayed sexual maturity in males; and reduced litter size, embryonic survival, and placental function in females. At the molecular level, zinc regulates the activity of enzymes (e.g., SOD), signaling pathways (MAPK, NF-κB), and transcription factors (MTF-1, Sp1) to maintain homeostasis. Both zinc deficiency (due to dietary insufficiency, malabsorption, or physiological stress) and zinc excess (from environmental pollution or feed oversupplementation) adversely affect health, disrupting mineral balance, enzyme function, and gut microbiota. In animal production, inorganic (zinc oxide, zinc sulfate) and organic (zinc methionine) sources of zinc increase growth, immunity, and productivity, although sustainable strategies are needed to mitigate environmental risks. Future research should focus on novel zinc formulations, precision nutrition, and interactions with gut microbiota to optimize livestock health and sustainable husbandry.

## Introduction

### Multifunctional roles of zinc in animals

Zinc, an essential trace element for animals, plays a pivotal role in maintaining health and normal physiological functions in animals. Since its discovery as an essential nutrient for animals in the twentieth century, an increasing number of studies have focused on its various functions in the animal body [1].

**Fig. 1:**
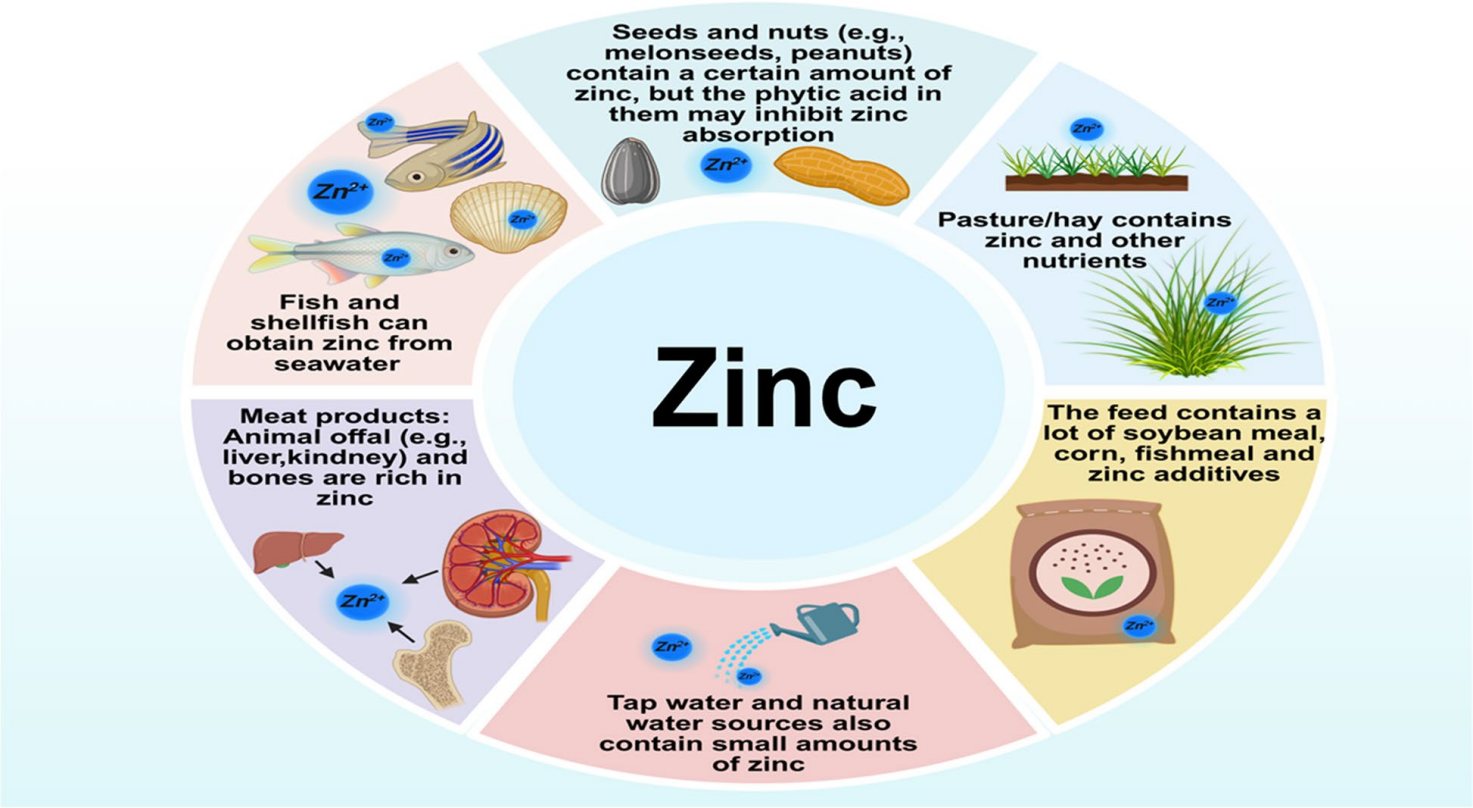
Sources of zinc ingested by animals

Zinc is widely present in the daily diet of animals. Animals can effectively absorb and store this key element by consuming zinc-rich feed ingredients. Common feed ingredients (e.g., meat meal, fish meal, grains, and nuts) are rich in zinc. In animal production, the zinc content in feed is carefully formulated to meet the requirements of carnivores and herbivores, while aquatic animals absorb mainly soluble zinc ions in water (Fig. 1). As feed is broken down in the digestive tract, zinc is absorbed into the bloodstream and is widely utilized throughout the body [2]. Zinc is involved in a wide range of physiological processes within animals. It serves as a crucial component of more than 300 enzymes and numerous functional proteins, participating in fundamental metabolic activities (e.g., DNA, RNA, protein, lipid, and vitamin metabolism) [3]. For example, zinc-containing enzymes (e.g., carbonic anhydrase) are essential for the efficient transport of carbon dioxide in the body, whereas DNA and RNA polymerases, which require zinc as a cofactor, are directly involved in genetic information transfer and protein synthesis, thereby significantly influencing cell division, growth, and tissue repair processes [4].

In terms of growth and development, zinc is highly important, especially for young animals. A sufficient supply of zinc is necessary for normal skeletal development. In chicks, zinc deficiency can lead to reduced long-bone osteogenic activity, decreased cartilage formation, and an abnormally increased amount of cartilage matrix, ultimately resulting in skeletal deformity [5, 6]. In addition, zinc has a profound effect on the reproductive performance of animals. In male animals, zinc deficiency can reduce the activity of various enzymes in the reproductive tract, leading to insufficient sex hormone secretion, stunted development of reproductive organs, atrophy of Sertoli cells and interstitial cells, and delayed sexual maturity [7, 8]. In female animals, zinc deficiency can cause symptoms (e.g., false estrus, reduced litter size, decreased zinc content in offspring, failure of fertilized egg implantation, and early embryonic death) [9, 10].

The immune system of animals also relies heavily on zinc. Zinc can regulate the development and function of immune organs, immune cells, and immune factors through zinc finger proteins and other forms. Zinc deficiency can cause immune organ atrophy and a significant reduction in the number of lymphocytes, thereby weakening an animal’s immune function and increasing its susceptibility to various diseases [11, 12]. For instance, in pigs, a lack of zinc can lead to a decrease in the activity of immune cells, increasing the animal’s vulnerability to pathogenic infections [13].

In the field of animal husbandry, the application of zinc has far-reaching importance. Rational use of zinc additives can improve animal growth performance, increase disease resistance, and reduce mortality, resulting in substantial economic benefits. For example, adding appropriate amounts of zinc to the diet of weaned piglets can effectively reduce the incidence of diarrhea, improve their immunity, and promote weight gain. However, improper use of zinc—either excessive or insufficient supplementation—can have adverse effects. Excessive zinc intake not only causes a waste of resources and increases breeding costs but also may lead to environmental pollution because of the large amounts of zinc excreted in animal feces. Insufficient zinc, on the other hand, can cause various deficiency symptoms in animals, affecting their growth and production performance [14, 15]. Therefore, a deep understanding of the relationship between zinc and animal health, as well as the underlying molecular regulatory mechanisms, is crucial for the development of the animal husbandry industry. Such knowledge can provide a scientific basis for the rational use of zinc in animal feed, improve animal production efficiency, and ensure the sustainable development of animal husbandry.

### Objective and outline

The objective of this review is to comprehensively summarize the current research status regarding the relationship between zinc and animal health as well as the underlying molecular regulatory mechanisms. This review aims to provide a theoretical basis for further research on zinc in animal nutrition and health, as well as practical guidance for the rational application of zinc in animal husbandry production. The main contents covered in this review include the following: First, the metabolism of zinc in animals, including its absorption, distribution, transport, and excretion, is elaborated on, and the factors that affect these processes are considered. Second, the effects of zinc on animal health, including its effects on growth and development, reproductive performance, immune function, and the normal functioning of various organs, are comprehensively expounded on. Third, the molecular regulatory mechanisms of zinc in animals, including how zinc participates in the regulation of gene expression through zinc finger proteins and other molecules and how it affects enzyme activity and signal transduction pathways at the molecular level to maintain normal physiological functioning, are explored. Finally, on the basis of the abovementioned content, future research directions in this field are anticipated, with the goal of promoting the continuous development of research on zinc and animal health.

## Zinc metabolism in animals

### Absorption of zinc

#### Absorption sites in different animals

**Fig. 2:**
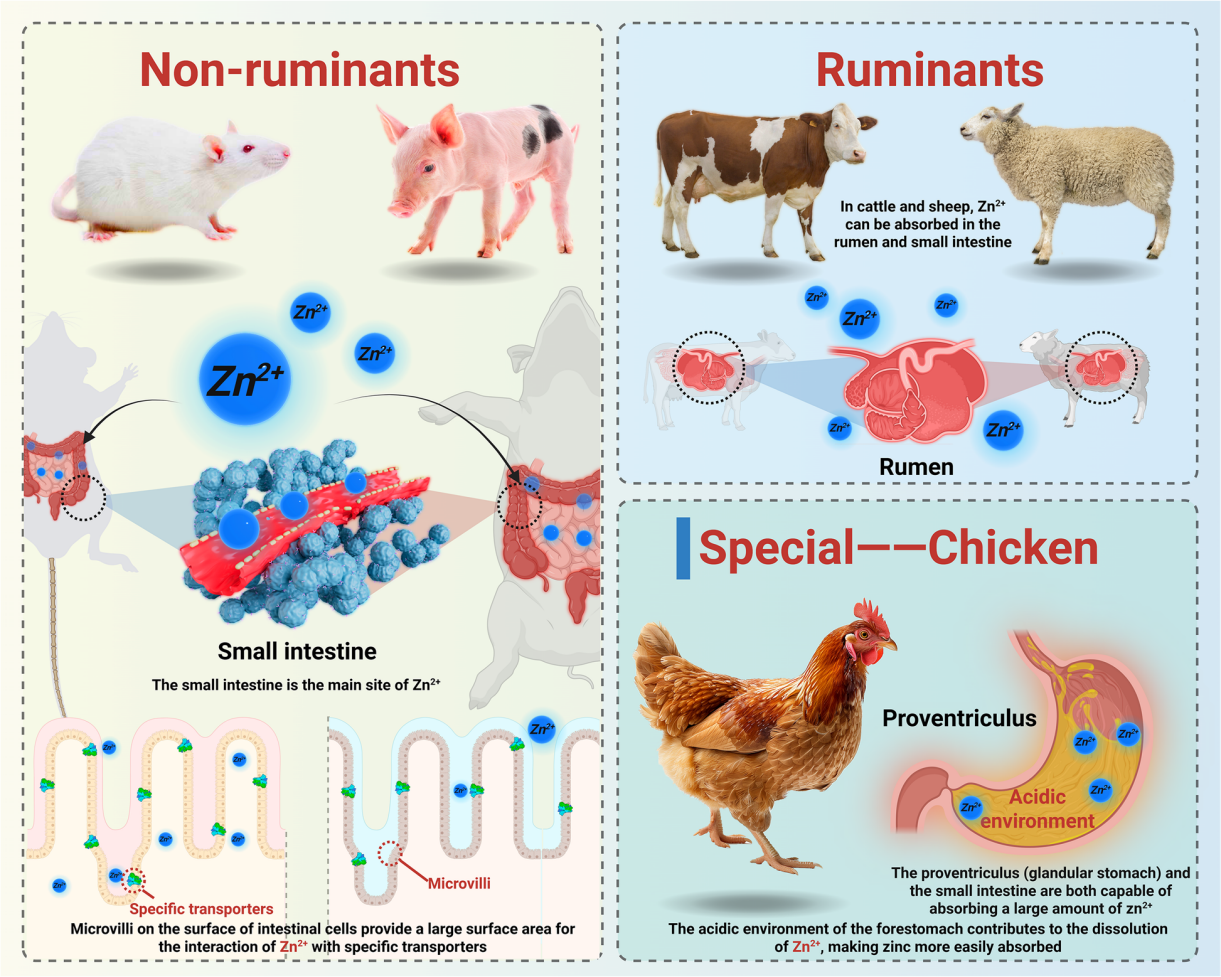
Different animals absorb zinc at different sites

The absorption of zinc in animals varies by species. In non-ruminants (e.g., pigs and rats), the small intestine is the primary site of zinc absorption. Specifically, the duodenum, jejunum, and ileum play crucial roles in zinc uptake [16]. For instance, in rats, a large amount of zinc is absorbed in the duodenum, where the microvilli on the surface of enterocytes provide a large surface area for zinc to interact with specific transporters [17] (Fig. 2).

In ruminants e.g., cattle and sheep, zinc can be absorbed in both the abomasum and the small intestine. Approximately one-third of dietary zinc is absorbed in the abomasum, which is the true stomach of ruminants, while the remainder is absorbed in the small intestine. The unique digestive physiology of ruminants, with their complex rumen fermentation process, may influence the availability of zinc for absorption in different parts of the digestive tract [18] (Fig. 2).

Poultry (e.g., chickens) have a different pattern. Both the proventriculus (glandular stomach) and the small intestine are capable of absorbing a large amount of zinc. The acidic environment in the proventriculus may help in the solubilization of zinc-containing compounds, making zinc more accessible for absorption [19]. Research has shown that in broiler chickens, the ileum may be the main site of zinc absorption, with its unique set of transporters and absorptive mechanisms contributing to efficient zinc uptake [20] (Fig. 2).

#### Factors affecting absorption

The absorption of zinc is influenced by multiple factors, including feed components, other elements, vitamins, the zinc source and the physiological state of the organism.

Feed ingredients substantially affect zinc absorption. Amino acids, especially histidine and cysteine, can form complexes with zinc, increasing its solubility and absorption. For example, compared with free zinc ions, histidine–zinc complexes are more easily absorbed in the intestine [21, 22]. Similarly, the source of zinc can affect its absorption by animals. Compared with inorganic zinc salts, organic complexed zinc is absorbed more easily, and its bioavailability is significantly increased [19]. In addition, phytic acid, which is abundant in grains and legumes, can bind strongly to zinc, forming insoluble complexes that reduce zinc bioavailability [23]. Dietary fiber, especially in large amounts, can also interfere with zinc absorption by physically entrapping zinc or competing for binding sites on the intestinal mucosa [24].

The presence of other elements in the diet can either promote or inhibit zinc absorption. Calcium, when present at high levels, can reduce zinc absorption. Consumption of high-calcium diets can lead to the formation of insoluble calcium‒zinc‒phosphate complexes in the intestine, decreasing the amount of zinc available for absorption [25]. Copper and iron, at certain ratios, can interact with zinc during the absorption process. Excessive copper can compete with zinc for binding to the same transport proteins, leading to reduced zinc absorption. Similarly, high levels of iron may also interfere with zinc uptake, as both zinc and iron share some common absorption pathways in intestinal cells [26].

Vitamins also play a role in zinc absorption. Vitamin D_3_ can increase zinc absorption, possibly by regulating the expression of zinc-binding proteins and transporters in the intestinal mucosa [27]. Vitamin A is also associated with zinc absorption, and deficiency of vitamin A may lead to reduced zinc utilization in the body [28].

The physiological state of the animal has a profound effect on zinc absorption. During growth, pregnancy, and lactation, animals have an increased demand for zinc, and the body responds by upregulating the expression of zinc transporters in the intestine, thereby increasing zinc absorption [10]. For example, in pregnant sows, the expression of zinc transport proteins in the small intestine is significantly increased to meet the needs of the developing fetus [29]. In contrast, in animals with gastrointestinal diseases (e.g., diarrhea), the integrity of the intestinal mucosa is damaged, possibly leading to reduced zinc absorption and increased zinc loss [30].

### Transport of zinc

Once absorbed into the animal bloodstream, zinc binds to plasma albumin, which serves as a major transport protein for zinc in the blood. This binding is crucial because it not only helps in the transport of zinc but also plays a role in maintaining the stability and solubility of zinc in the blood. Approximately 70%–80% of the zinc in plasma is bound to albumin, while the remainder is associated with other proteins or exists in a free ionic form. The zinc–albumin complex is then transported to various tissues and organs through the circulatory system [31].

The distribution of zinc in different tissues is regulated in animals. Different tissues have varying requirements for zinc, and the body adjusts the delivery of zinc accordingly [32]. High-metabolic-activity tissues (e.g., the liver, pancreas, and kidneys) tend to accumulate more zinc. The liver, in particular, serves as an important organ for zinc storage and metabolism. Zinc can be taken up by liver cells through specific zinc transporters and is involved in many enzymatic reactions and metabolic pathways within the liver [33].

In addition to its transport in the general circulation, zinc has a specific transport mechanism in the placenta during pregnancy. Zinc is essential for the normal development of the fetus, and its transfer across the placenta is a tightly regulated process. The placenta contains specific zinc transporters, including zinc transporter 1 (ZnT1), zinc transporter 2 (ZnT2), zinc transporter 3 (ZnT3), and zinc transporter 4 (ZnT4), which play crucial roles in the transfer of zinc from the maternal circulation to the fetal circulation. ZnT1 is involved in the efflux of zinc from the placenta to the fetal side, ensuring an adequate supply of zinc for the developing fetus. ZnT2 is located mainly in the apical membrane of placental syncytiotrophoblasts and may participate in the uptake of zinc from the maternal circulation. ZnT3 is expressed in the placenta and is thought to be involved in the storage and release of zinc in specific vesicles within placental cells. ZnT4 also contributes to the transfer of zinc across the placenta, and its expression level can be regulated by factors e.g., the maternal zinc status and hormonal changes during pregnancy [34–36].

Research has shown that the transfer of zinc across the placenta is a saturable process, indicating that the amount of zinc that can be transferred is limited, even when the maternal zinc level is high. This is an important regulatory mechanism to protect the fetus from excessive zinc exposure [37]. Moreover, any disruption in the function of these zinc transporters or changes in the maternal–fetal environment can affect the normal transfer of zinc across the placenta, potentially leading to adverse effects on fetal development. For instance, maternal zinc deficiency during pregnancy can reduce the expression of placental zinc transporters, resulting in insufficient zinc supply to the fetus, which may lead to growth retardation, congenital malformations, and other developmental disorders [38, 39].

### Storage and metabolism of zinc

**Fig. 3:**
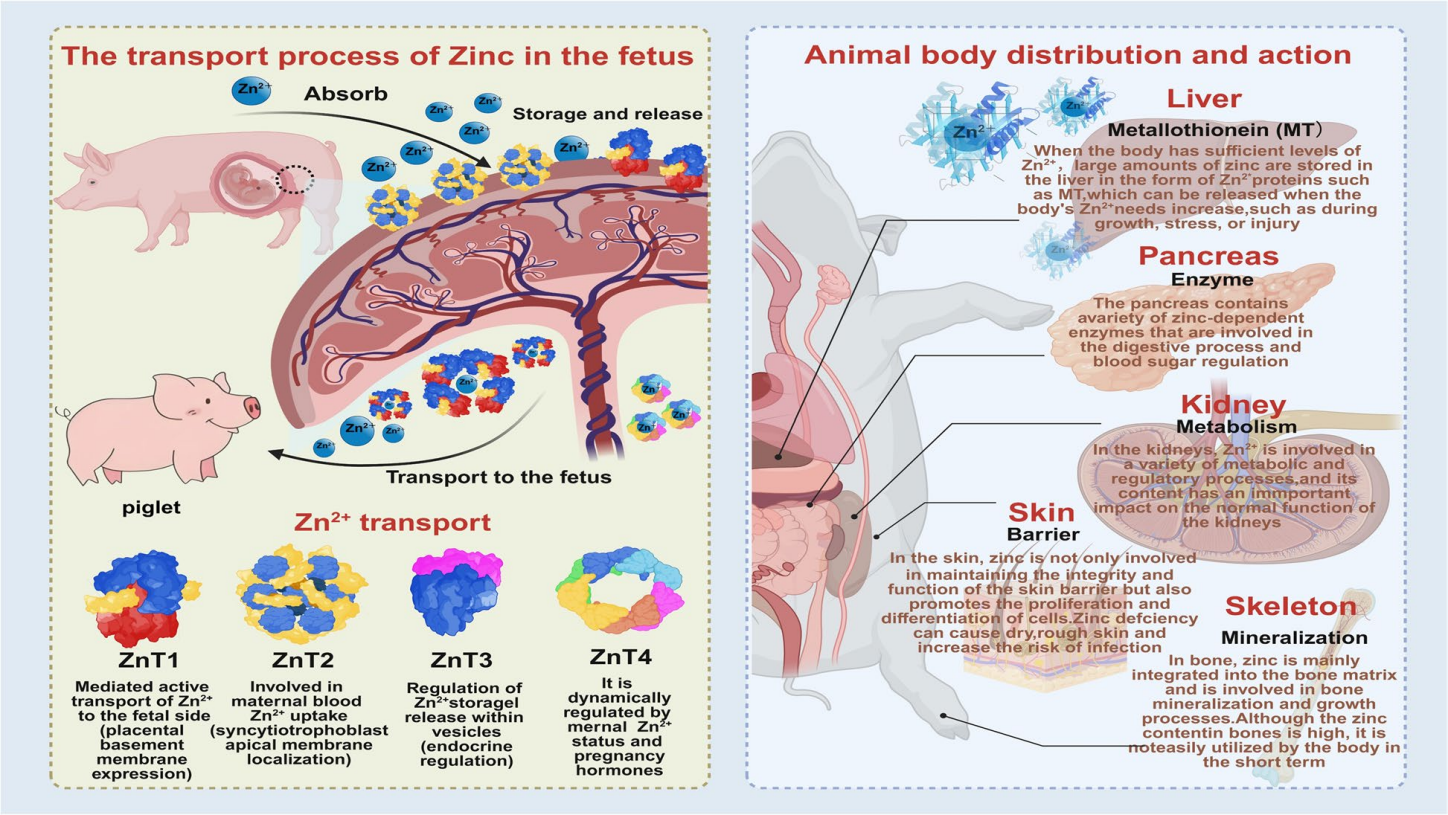
The transport of zinc in the body and its effects on various tissues and organs (using pigs as a template)

Following its absorption, zinc is transported via the bloodstream and distributed to various tissues, where its storage and metabolic turnover rates significantly vary on the basis of the specific physiological functions of the tissue [40] (Fig. 3).

The liver is the central hub for zinc storage and systemic homeostasis. It quickly absorbs zinc from the portal circulation and stores it through its binding to metallothionein (MT). MT is a cysteine-rich protein that can bind to zinc, helping to maintain the intracellular zinc balance. This zinc pool can act as a readily mobilizable reserve, releasing zinc whenever there is an increased physiological need, e.g., during growth, stress, or infection [33, 37]. Zinc is actively accumulated by tissues with high metabolic and secretory activity to support their functions, as exemplified by the pancreas and kidneys. The pancreas requires zinc for the production and secretion of insulin and digestive enzymes [41]. In the kidneys, zinc is involved in tubular reabsorption and various metabolic processes, and its deficiency can impair kidney function [42].

In contrast, tissues with structural or long-term functions exhibit slower zinc turnover. Zinc is incorporated into the hydroxyapatite matrix of bone during mineralization. Although bone contains a substantial portion of the total body zinc reserve, its mobilization from bone is slow and not regulated primarily for short-term zinc homeostasis [6]. Similarly, nervous system-localized zinc, particularly in synaptic vesicles, is involved in neurotransmission and is relatively stable, reflecting its role in long-term neuronal signaling and plasticity [43].

Other organs also play important roles. Owing to its large mass, skeletal muscle is a major reservoir of zinc, albeit at a moderate concentration [44]. Skin and its appendages (e.g., hair and wool) require zinc to maintain structural integrity and cell proliferation; zinc deficiency often manifests as skin lesions and poor hair quality [45, 46]. The reproductive organs (testes and ovaries) maintain relatively high levels of zinc that are crucial for gametogenesis and hormone synthesis [47].

The dynamic distribution of zinc is tightly regulated by a network of zinc transporters (ZnT and ZIP families) that control zinc influx, efflux, and intracellular compartmentalization across these tissues, ensuring that each organ maintains a zinc level appropriate for its function [7].

### Excretion of zinc

After zinc is metabolized, its excretion from animals occurs mainly through feces. A large amount of zinc is excreted in the form of bile, pancreatic juice, and other digestive juices. These digestive juices are secreted into the digestive tract and are then excreted with the feces [15, 48]. For example, in pigs, a significant portion of endogenous zinc is excreted in the feces via digestive juices. The excretion of endogenous zinc through feces is an important mechanism for the animal to maintain zinc homeostasis. When the body has an excessive supply of zinc, more zinc is excreted through this pathway to prevent zinc accumulation [49].

In addition to normal digestive system-related excretion, production animals excrete zinc with their products. Dairy cows excrete a certain amount of zinc in milk during lactation. The amount of zinc present in milk can vary substantially, with the excretion process largely dictated by the cow's diet and overall zinc status. The normal dietary zinc requirement for dairy cows is 60 mg/kg, and during the lactation period, it increases to 100 mg/kg, which can significantly improve mammary gland health and the sustainability of milk production. However, the concentration of zinc in the milk produced will also increase [50, 51]. Similarly, in laying hens, the recommended feed zinc level is 35 mg/kg, excess zinc will be excreted in the eggs [52]. Zinc in eggs is essential for the development of the embryo. The amount of zinc excreted with eggs is related to the hen's zinc intake and its ability to transfer zinc to the egg [53, 54]. The excretory pattern of zinc in production animals has important implications for animal production and the quality of animal-derived products. Understanding these excretion characteristics can aid in the formulation of appropriate feeding strategies to ensure both the health of the animals and the quality of the products they produce.

## The roles of zinc in animal physiology and health

### Growth and development

#### Role in enzyme-mediated metabolic processes

Zinc plays a fundamental role in growth and development by participating in enzyme-mediated metabolic processes in animals. It is an essential component of numerous enzymes that are involved in the metabolism of carbohydrates, lipids, proteins, and nucleic acids [1].

Animals contain large amounts of carbonic anhydrase. This zinc-containing enzyme catalyzes the reversible hydration of carbon dioxide to form bicarbonate and protons, a reaction crucial for maintaining acid‒base balance and facilitating efficient carbon dioxide transport in the respiratory system. The reaction is as follows:

$${\text{CO}}_2+{\text{H}}_2\text{O}\rightleftharpoons{\mathrm{HCO_3}}^-+\text{H}^+$$

Zinc deficiency impairs carbonic anhydrase activity, disrupting carbon dioxide metabolism and affecting energy production and waste removal processes [55, 56]. Conversely, excessive zinc intake may impair enzyme function. Excessive zinc absorption induces copper deficiency through competitive absorption, damaging copper-dependent enzymes in the respiratory chain, including cytochrome C oxidase. This metabolic disruption may secondarily affect carbonic anhydrase function by altering acid‒base equilibrium [26, 57].

Hydroxy peptidases are zinc-dependent enzymes critical for protein digestion, as they cleave peptide bonds at the N-terminus of peptides to release amino acids. In the small intestine, their proper function is essential for breaking down dietary proteins into absorbable peptides and amino acids, which support tissue repair, growth, and the synthesis of proteins, hormones, and enzymes. Zinc deficiency reduces hydroxy peptidase activity, impairing protein digestion and limiting the supply of essential amino acids, thereby hindering muscle development and immune function. Conversely, while excessive zinc does not directly inhibit these enzymes, it can diminish overall proteolytic efficiency by damaging the intestinal mucosa or disrupting the gut microbiota, ultimately compromising nutrient absorption and digestive processes [58].

Zinc-containing DNA and RNA polymerases play crucial roles in nucleic acid metabolism. DNA polymerases ensure accurate DNA replication during cell division, whereas RNA polymerases catalyze DNA transcription for protein synthesis. Adequate zinc is essential for maintaining the stability and catalytic activity of these enzymes. Zinc deficiency disrupts DNA replication and transcription, resulting in aberrant gene expression that compromises cell division, growth, and development [3]. These effects are especially pronounced in highly proliferative tissues—including embryonic cells and growth plates—and may lead to developmental abnormalities or cell death. However, excessive zinc may disrupt the precise coordination of zinc ions within polymerase active sites, reducing replication and transcription fidelity, increasing mutation rates, and ultimately inhibiting cell growth and function [59].

#### Influence on bone development

Zinc is highly important for bone development in animals. Zinc deficiency can lead to various skeletal abnormalities. In chicks, zinc deficiency has been shown to reduce the activity of osteoblasts, the cells responsible for bone formation. Specifically, long bone osteogenic activity is decreased, meaning that the growth and mineralization of long bones are impaired. This can result in decreased length and strength of long bones. Additionally, the formation of cartilage in the growth plates is affected. There is an abnormal increase in the amount of cartilage matrix, but the normal process of cartilage ossification is disrupted. Thus, chicks may exhibit skeletal deformities, including bowed legs or stunted growth [5].

In calves, zinc deficiency can cause joint-related problems. The joints may become swollen and stiff, which affects the mobility of the calf [60]. This effect is due to the role of zinc in maintaining the normal structure and function of the connective tissues in joints [61]. Zinc is involved in the synthesis of collagen and other extracellular matrix components that are essential for the integrity and flexibility of joints. When zinc is deficient, the production of these components is disrupted, leading to joint abnormalities [62].

Research on the impact of zinc deficiency on bone development in animals has provided valuable insights into the importance of zinc in maintaining skeletal health. These findings have practical implications for animal husbandry, as ensuring an adequate supply of zinc in the diet of livestock can help prevent skeletal disorders and promote healthy growth and development.

### Immune function

#### Effects on immune organs and cells

Zinc plays a crucial role in maintaining the normal function of the immune system in animals. Zinc deficiency can lead to the atrophy of immune organs [11]. In laboratory-raised mice fed a zinc-deficient diet, the thymus, which is a primary immune organ, shows significant atrophy. The thymus is responsible for the maturation and differentiation of T lymphocytes. A decrease in the size of the thymus due to zinc deficiency leads to a reduction in the production of mature T lymphocytes, which are essential for cell-mediated immunity [63, 64].

Zinc deficiency also markedly decreases the populations of lymphocytes. These critical immune cells—encompassing T lymphocytes and B lymphocytes—play central roles in adaptive immunity. T lymphocytes drive cell-mediated responses, including the elimination of virus-infected and cancerous cells, whereas B lymphocytes facilitate humoral immunity by producing antibodies that target and neutralize invading pathogens [65]. In pigs with zinc deficiency, the number of lymphocytes in the peripheral blood and lymphoid tissues decreases, resulting in a weakened immune response [66].

Zinc is also involved in regulating the functions of immune cells. For example, in macrophages, zinc is required for phagocytic activity. Macrophages are large immune cells that can engulf and digest pathogens. Adequate zinc levels ensure that macrophages can efficiently recognize, engulf, and kill bacteria and other foreign invaders. When zinc is deficient, the phagocytic ability of macrophages is impaired, reducing the ability of the animal to clear infections [67, 68].

Natural killer (NK) cells, another type of immune cell, also rely on zinc for their normal function. NK cells can directly kill virus-infected cells and tumor cells without prior sensitization. Zinc deficiency can lead to a decrease in the cytotoxic activity of NK cells, increasing the susceptibility of animals to viral infections and tumor development [69, 70].

In addition, zinc is essential for the development of immune factors [71]. Cytokines, including interleukin-1 (IL-1), interleukin-2 (IL-2), and tumor necrosis factor-alpha (TNF-α), are important immune factors. IL-1 is involved in the activation of T lymphocytes and the initiation of the immune response. IL-2 promotes the proliferation and activation of T lymphocytes and NK cells. TNF-α can induce apoptosis in tumor cells and play a role in the inflammatory response. Zinc deficiency can disrupt the production and function of these cytokines, affecting the overall immune response of the animal [72, 73].

Conversely, excessive zinc intake can also impair immune function. Excessive zinc consumption may disrupt the balance of immune cell populations and cytokine production. For example, high zinc levels have been shown to suppress lymphocyte proliferation and alter the Th1/Th2 cytokine balance, leading to inappropriate immune responses [74]. In pigs, dietary zinc intake far exceeding the requirements reduces NK cell numbers and macrophage activity, mimicking the effects of zinc deficiency—indicating a narrow optimal range for zinc-mediated immunoregulation [66]. Furthermore, excessive zinc may induce oxidative stress and inflammatory responses, further disrupting immune homeostasis.

#### Immune-related molecular mechanisms

At the molecular level, zinc exerts its regulatory effects on the immune function of animals mainly through zinc finger proteins and other forms [75]. Zinc finger proteins are a class of proteins that contain zinc-binding domains and can specifically bind to DNA, RNA, or other proteins. Zinc finger proteins play crucial roles in the regulation of gene transcription [76].

In the animal immune system, zinc finger proteins are involved in the regulation of the expression of immune-related genes. For example, some zinc finger proteins can bind to the promoter regions of cytokine genes (e.g., *IL-2*). By binding to the promoter, they can either promote or inhibit the transcription of the gene. In the presence of sufficient zinc, zinc finger proteins can maintain their proper conformation and function, accurately regulating the expression of cytokine genes [77, 78]. When zinc is deficient, the structure and function of zinc finger proteins may be disrupted, leading to abnormal regulation of cytokine gene expression. This can result in either excessive or insufficient production of cytokines, both of which can disrupt the normal immune response [73].

Zinc also affects the translation of immune-related proteins. It can interact with ribosomes and translation-related factors to ensure the accurate translation of mRNA into proteins. In the synthesis of immunoglobulins, which are key proteins in the humoral immune response, zinc is necessary for the proper folding and assembly of immunoglobulin chains. In the absence of sufficient zinc, the synthesis of immunoglobulins may be impaired, leading to a decrease in antibody production and weakened humoral immunity [79].

Moreover, zinc can regulate the activity of some enzymes involved in the immune response through allosteric regulation. For example, protein kinases and phosphatases are important enzymes in signal transduction pathways within immune cells. Zinc can bind to these enzymes and change their conformation, thereby regulating their catalytic activity [80, 81]. In the T cell receptor signaling pathway, zinc-mediated regulation of protein kinases and phosphatases can affect the activation, proliferation, and differentiation of T cells, ultimately influencing the cell-mediated immune response [77].

**Fig. 4:**
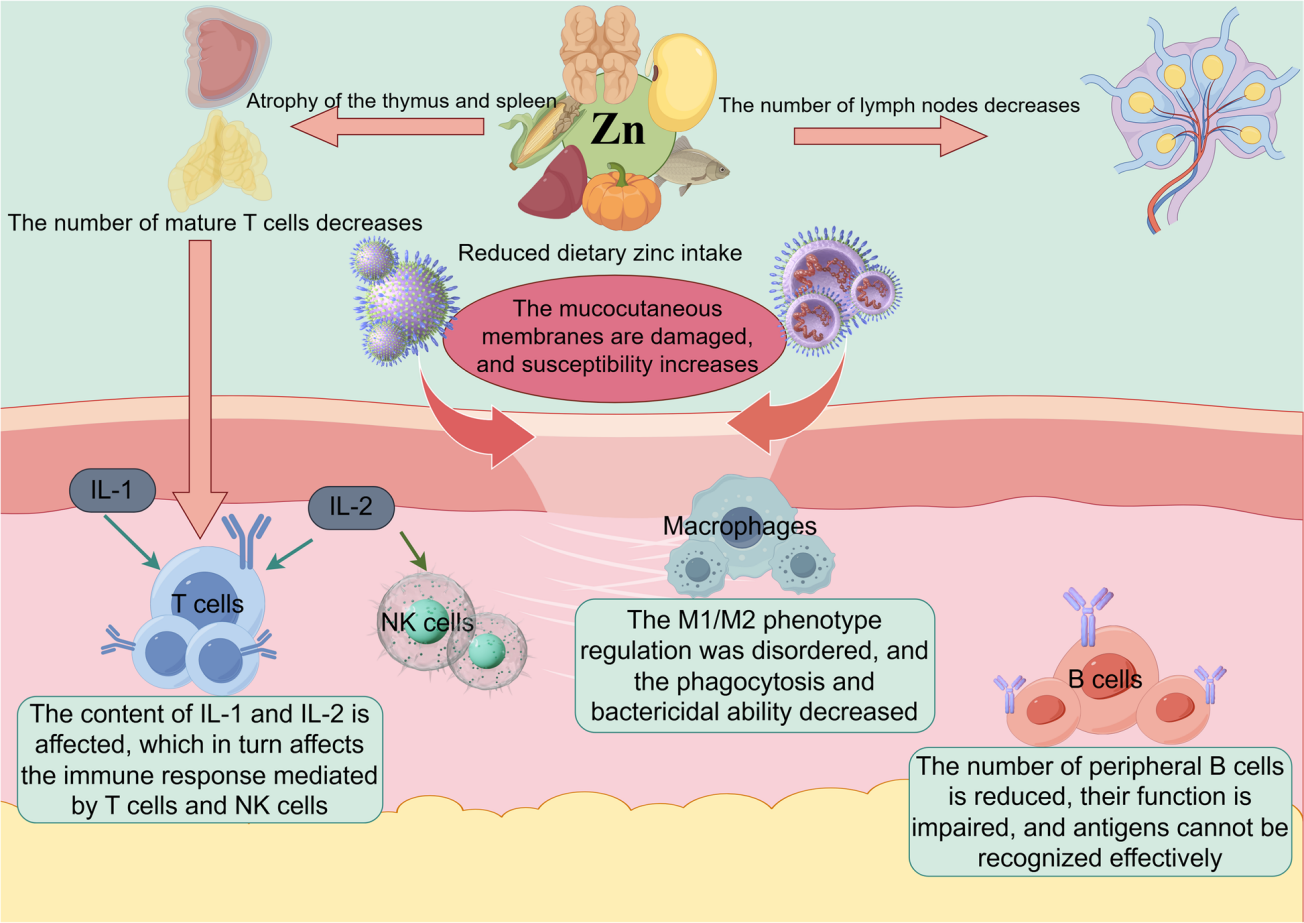
Regulatory roles of zinc in the immune system

At high concentrations, zinc may overactivate or inhibit these molecular pathways. For instance, excess zinc can trigger chronic inflammation through the production of excessive proinflammatory cytokines through uncontrolled nuclear factor-kappa B (NF-κB) activation [42]. Furthermore, excessive zinc disrupts zinc finger protein function by saturating binding sites, leading to protein misfolding and loss of DNA-binding specificity, which may further dysregulate immune gene expression. Thus, both deficiency and excess of zinc can disrupt the delicate molecular mechanisms that regulate immune function [69] (Fig. 4).

### Reproductive performance

#### Male reproductive system

Zinc is highly important for the normal development and function of the male reproductive system in animals. In male animals, zinc deficiency can lead to a series of adverse effects [8].

Zinc deficiency leads to a significant decrease in the activity of enzymes in the reproductive tract of male animals. For example, the activity of alkaline phosphatase, which is crucial for the normal physiological functions of male reproductive organs, is closely related to zinc levels. This enzyme is involved in numerous metabolic processes within the reproductive tract, including the hydrolysis of phosphate esters, which in turn supplies energy and substrates essential for reproductive development and function. Zinc deficiency can lead to reduced alkaline phosphatase activity, thereby disrupting these vital metabolic pathways [82–84]. Zinc deficiency also affects the activity of other enzymes, including lactate dehydrogenase and hyaluronidase, which are important for sperm production, maturation, and motility. Lactate dehydrogenase is involved in energy metabolism in sperm cells, and a reduction in its activity can lead to an insufficient energy supply for sperm, affecting their motility and viability [85]. Hyaluronidase is necessary for sperm to penetrate the egg during fertilization, and its reduced activity due to zinc deficiency can impede the fertilization process [86].

The secretion of sex hormones is also insufficient in zinc-deficient male animals. Testosterone, the primary male sex hormone, plays a vital role in the development of male reproductive organs, spermatogenesis, and the maintenance of the secondary sex characteristics of males [87, 88]. Zinc deficiency can disrupt the normal function of the hypothalamus–pituitary–gonadal axis, which is responsible for regulating the secretion of sex hormones. This disruption leads to a decrease in the secretion of luteinizing hormone (LH) and follicle-stimulating hormone (FSH) from the pituitary gland. LH stimulates Leydig cells in the testes to produce testosterone, while FSH is involved in spermatogenesis. A decrease in the levels of these hormones due to zinc deficiency results in reduced testosterone production, which in turn affects the development and function of the male reproductive system [89].

Zinc deficiency can cause abnormal development of the reproductive organs in male animals. The testes, the main reproductive organ in male animals, are capable of producing sperm, and a decrease in zinc intake can lead to abnormal testicular development in animals. The seminiferous tubules in the testes, where spermatogenesis occurs, may undergo atrophy. Sertoli cells, which provide support and nutrition for developing sperm cells, and interstitial cells, which produce testosterone, also undergo atrophy. This atrophy leads to a reduction in sperm production and quality [90, 91]. For example, zinc deficiency leads to a reduction in spermatozoa production and an increase in morphological abnormalities, notably defects in head and tail structure. The maturation process of sperm is also disrupted, resulting in an increased percentage of immature sperm in the semen [92]. Moreover, the development of accessory reproductive organs (e.g., the prostate gland and seminal vesicles) can be impaired by zinc deficiency. These organs play important roles in the production of seminal fluid, which provides nutrients and a suitable environment for sperm. Abnormal development of these organs due to zinc deficiency can lead to changes in the composition and quality of seminal fluid, further affecting sperm function and fertility [93].

**Fig. 5:**
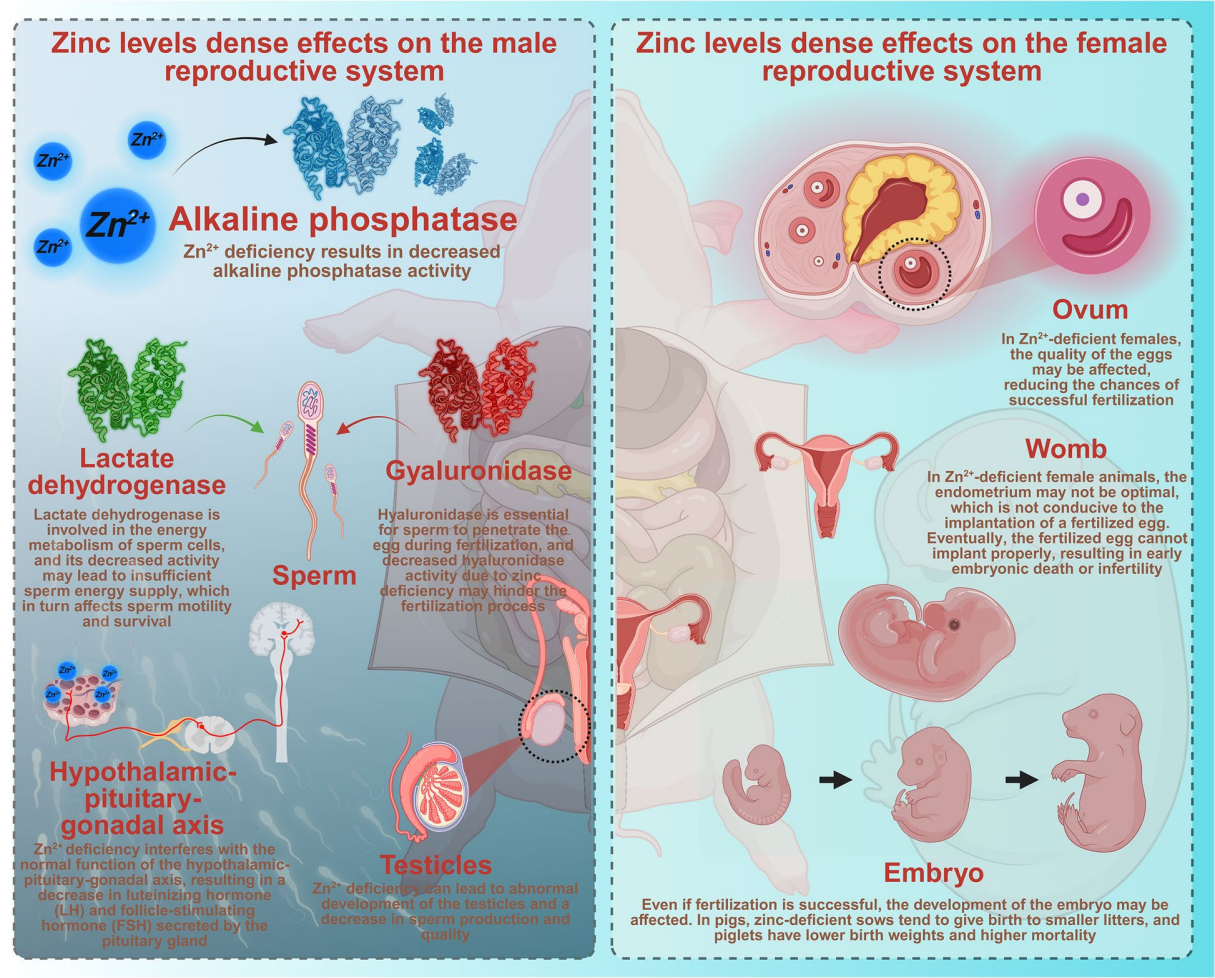
Effects of zinc on the reproductive system in male and female animals (using pigs as a model)

The sexual maturity of male animals is often delayed in the presence of zinc deficiency. Zinc is essential for the normal development and function of the endocrine system, which regulates sexual maturation. In young male animals, zinc deficiency can slow the development of the hypothalamus‒pituitary‒gonadal axis, delaying the onset of puberty. This delay can have long-term consequences for the animal’s reproductive performance, as it may reduce the number of breeding opportunities and the overall reproductive efficiency [88, 92] (Fig. 5). In livestock production, a delay in sexual maturity can increase the time and cost required to raise animals to a breeding-ready state, affecting the economic benefit to the farm.

#### Female reproductive system

Zinc deficiency in female animals can cause multiple problems related to reproductive performance. One of the common symptoms is false estrus. The normal estrous cycle in female animals is regulated by a complex hormonal system, and zinc plays an important role in maintaining the normal function of this system. In zinc-deficient female animals, the hormonal balance is disrupted, leading to abnormal estrous behavior. False estrus is a reproductive condition characterized by the exhibition of typical estrus behaviors, including heightened sexual receptivity, despite the absence of ovulation. This state can lead to incorrect mating decisions, wasting breeding opportunities and potentially reducing the overall reproductive efficiency of the herd or flock.

The litter size of female animals is often reduced when they are zinc deficient [94]. Zinc is involved in various processes related to pregnancy and embryo development. During fertilization, zinc is necessary for the normal function of sperm and egg cells. In zinc-deficient females, the quality of the eggs may be affected, reducing the likelihood of successful fertilization. Even if fertilization occurs, the development of the embryo may be impaired. Zinc is required for the normal division and differentiation of embryonic cells. A lack of zinc can lead to abnormal cell division, resulting in developmental defects in the embryo [95]. For example, zinc-deficient sows often produce smaller litters, and the piglets may have lower birth weights and higher mortality rates [21].

The implantation of fertilized eggs may be abnormal in zinc-deficient female animals. After fertilization, the fertilized egg needs to implant into the uterine wall to establish a pregnancy. Zinc is involved in the regulation of the uterine environment and the interaction between the embryo and the uterine tissue. In zinc-deficient females, the uterine lining may not be in an optimal state for implantation. The expression of certain adhesion molecules and cytokines that are important for implantation may be affected by zinc deficiency. This can lead to the failure of fertilized eggs to implant properly, resulting in early embryonic death or infertility [96, 97]. In cattle, zinc deficiency is a major cause of early pregnancy loss, often resulting from implantation failure [94].

Zinc deficiency can also lead to problems during pregnancy, including miscarriage and impaired fetal development. Zinc is essential for the normal development of the placenta, which is responsible for providing nutrients and oxygen to the developing fetus. In zinc-deficient pregnant animals, the placenta may not develop properly, leading to insufficient nutrient supply to the fetus. This can cause growth retardation, congenital malformations, and an increased risk of miscarriage [98]. When pregnant cows experience zinc deficiency, their calves may develop skeletal deformities and have a weakened immune system. This impairment results in a heightened susceptibility to various diseases postnatally [99] (Fig. 5).

### Other physiological functions

#### Vision system

Zinc plays a crucial role in maintaining normal visual function in animals. It is involved in the metabolism of vitamin A, which is essential for vision. Zinc, a key component of retinol dehydrogenase, plays a decisive role in animal visual function [100, 101]. The absorption and metabolism of vitamin A vary significantly across animal species. Ruminants like cattle and sheep are particularly vulnerable to zinc-deficiency-induced visual disturbances, notably sluggishness at dusk, because their rumen microorganisms absorb zinc less efficiently [102, 103]. Compared with ruminants, pigs have a higher absorption rate of zinc. Therefore, zinc can maintain the homeostasis and photoreceptive function of retinal structures and regulate the adaptability of pigs to light [104]. Animal experiments have shown that zinc directly affects the dark vision ability, predation efficiency and survival adaptability of animals by regulating retinaldehyde metabolism.

In addition to playing a role in vitamin A metabolism, zinc is important for maintaining the normal structure and function of eye tissues in animals. The retina, which is the light-sensitive layer at the back of the eye, contains a high concentration of zinc. Zinc helps maintain the integrity of retinal cells and their ability to respond to light stimuli. It also plays a role in protecting the retina from oxidative damage. In the presence of light, the retina is exposed to a high level of oxidative stress, and zinc, as an antioxidant, can help to neutralize free radicals and prevent damage to retinal cells [105, 106].

Research has shown that zinc deficiency in animals can lead to abnormal retinal function. In zinc-deficient rats, changes in the electroretinogram (ERG), which is a test that measures the electrical response of the retina to light, have been detected. The ERG results revealed a decrease in the b-wave amplitude, which is an indication of abnormal function of retinal bipolar cells and Müller cells. These changes in the ERG are associated with a reduction in visual acuity and an increased susceptibility to retinal damage [107]. Zinc deficiency may also induce ocular dysfunction in animals; a common manifestation is night blindness, which results from the disruption of retinal vitamin A metabolism [100].

#### Cardiovascular system

Zinc has a substantial effect on the cardiovascular system of animals, mainly through its ability to inhibit lipid peroxidation and histamine release, maintain vascular wall permeability, and play a role in stabilizing cell and lysosomal membranes.

Lipid peroxidation is a process in which free radicals attack and oxidize lipids in cell membranes, leading to membrane damage and the production of harmful byproducts. Zinc can act as an antioxidant to inhibit this process. It can scavenge free radicals (e.g., superoxide anions and hydroxyl radicals), reducing their ability to initiate lipid peroxidation. By inhibiting lipid peroxidation, zinc helps maintain the integrity and normal function of cell membranes in the cardiovascular system [108, 109]. Zinc also maintains the normal permeability of blood vessel walls in animals and ensures the integrity of the endothelial barrier by participating in the structural development and functional regulation of vascular endothelial cells. Specifically, by acting as an essential cofactor for numerous enzymes, zinc facilitates the synthesis and stabilization of critical structural proteins—particularly endothelial tight junction proteins. This action strengthens intercellular connections and thereby mitigates the pathological leakage of vascular components (e.g., fluid and proteins) into the interstitial space. Moreover, zinc can inhibit the excessive release of proinflammatory factors in animals; if these factors are present in large quantities, they can damage the structure of endothelial cells, destroy the normal barrier function of the blood vessel wall, and lead to increased permeability [110–112]. Therefore, an adequate supply of zinc plays a nonnegligible role in maintaining the normal permeability of blood vessel walls in animals, ensuring proper blood circulation and internal environmental stability.

Zinc also plays a role in inhibiting histamine release. Histamine is a chemical mediator that is released during allergic reactions and inflammation. Excessive release of histamine can cause vasodilation, increased vascular permeability, and smooth muscle contraction in the cardiovascular system, which can disrupt normal cardiovascular function. Zinc can interact with mast cells, the cells that store and release histamine, and inhibit the release of histamine. Through this mechanism, zinc stabilizes the cardiovascular system and prevents excessive inflammatory responses, thereby safeguarding the blood vessels and heart from potential damage [113].

Moreover, zinc is important for stabilizing cell membranes and lysosomal membranes. The integrity of cell membranes in the cardiovascular system, including those of cardiomyocytes (heart muscle cells) and endothelial cells, needs to be maintained to ensure normal function. Zinc can bind to membrane-associated proteins and lipids, increasing membrane stability. Lysosomal membranes, which surround lysosomes (organelles that contain digestive enzymes), also require zinc for stability. In the case of myocardial infarction (heart attack) or other forms of heart injury, the integrity of cell membranes and lysosomal membranes is crucial. When the heart is damaged, lysosomal enzymes can be released if lysosomal membranes are unstable. Zinc helps prevent the premature release of these enzymes, reducing the extent of tissue damage during heart-related events [114]. In experimental models of myocardial ischemia‒reperfusion injury (during which the heart muscle is deprived of blood supply and then reperfused), damage to heart tissue is less severe in animals with sufficient zinc levels than in those with low zinc levels. The presence of zinc helps maintain the normal function of the heart under these stressful conditions, highlighting its importance for cardiovascular health [115, 116].

Therefore, in animal husbandry, scientifically based and reasonable supplementation with zinc can effectively maintain the homeostasis of the cardiovascular system of animals in a variety of ways, thereby substantially improving animal health, production performance and the economic benefits of breeding.

## Molecular regulatory mechanisms of zinc in animal health

### Zinc-related enzymes and their regulatory roles

#### Catalytic and regulatory functions of zinc-containing enzymes

Zinc-containing enzymes play diverse and crucial catalytic and regulatory roles in the animal body, with superoxide dismutase (SOD) being a prime example. Notably, zinc plays a direct and indispensable structural and catalytic role specifically in the Cu/Zn-SOD isoform. SOD is an antioxidant enzyme that exists in different isoforms in animals, including Cu/Zn-SOD, Mn-SOD, and Fe-SOD, each with its own tissue-specific distribution. Cu/Zn-SOD is located mainly in the cytoplasm of eukaryotic cells, Mn-SOD is found in mitochondria, and Fe-SOD is present in some prokaryotes and specific plant cell compartments. The primary function of SOD is to catalyze the dismutation of superoxide anions (${\mathrm O_{2}\bullet}^-$) into molecular oxygen (O_2_) and hydrogen peroxide (H_2_O_2_), as described by the following reaction:

$${\mathrm O_{2}\bullet}^-+2\mathrm H^+\xrightarrow{\mathrm{SOD}}{\mathrm O}_2+{\mathrm H}_2{\mathrm O}_2$$

This reaction is highly important for maintaining the balance between oxidative and antioxidative processes within cells. Superoxide anions are potent reactive oxygen species (ROS) that can cause significant damage to cellular components, including DNA, proteins, and lipids, if not properly scavenged. By converting superoxide anions into less reactive substances, SOD protects cells from oxidative stress [117, 118].

In the context of animal health, SOD-mediated antioxidant defense is essential for maintaining normal cellular metabolism. For example, in the liver, which is constantly exposed to various xenobiotics and oxidative stressors during detoxification processes, SOD activity helps to prevent damage to hepatocytes. A study on rats revealed that when they were exposed to oxidative stress induced by a high-fat diet, the activity of SOD in the liver decreased, leading to increased lipid peroxidation and liver cell damage. However, when the rats received zinc supplementation, the activity of Cu/Zn-SOD increased, effectively reducing oxidative damage and protecting the liver from injury [119, 120].

SOD also plays a critical role in the immune system. Immune cells (e.g., macrophages and neutrophils) generate superoxide anions as part of their antibacterial and antiviral defense mechanisms. However, excessive production of superoxide anions can also damage these immune cells. SOD in immune cells helps maintain a proper ROS balance, ensuring that the immune response is effective while minimizing self-damage [121]. In zinc-deficient animals, the activity of SOD in immune cells is often reduced, leading to impaired immune function and increased susceptibility to infections [122].

#### Regulation of gene expression by zinc-related enzymes

Zinc-related enzymes can influence gene expression, thereby regulating various cell physiological processes. One well-studied example is the role of zinc-containing transcription factors in regulating the expression of genes related to antioxidant defense. Metal-regulatory transcription factor 1 (MTF-1) is a zinc finger protein that can bind to metal-response elements (MREs) in the promoter regions of target genes. When the intracellular zinc concentration increases, zinc ions bind to MTF-1, causing a conformational change that enables MTF-1 to translocate to the nucleus and bind to MREs. This binding can then activate the transcription of genes encoding proteins involved in zinc homeostasis, including *MT* [123]. In addition to its role in zinc homeostasis, MT has antioxidant properties, as it can scavenge free radicals.

The regulation of *MT* gene expression by zinc-related factors is a complex process. In the case of zinc-containing enzymes, their activity can affect the overall cellular redox state, which in turn can influence the activation of MTF-1. For example, SOD-mediated reduction of superoxide anions can lead to a decrease in the activity of oxidative stress-related signaling pathways. This can stabilize MTF-1 and increase its binding to MREs, resulting in increased *MT* gene expression [34, 37].

Moreover, zinc-related enzymes can regulate the expression of genes involved in other physiological processes [75]. For instance, some zinc-containing proteases are involved in the processing of transcription factors. These proteases can cleave inactive forms of transcription factors, converting them into their active forms, which can then bind to DNA and regulate gene expression. In the context of cell growth and differentiation, zinc-containing enzymes may regulate the expression of genes encoding growth factors, cytokines, and cell cycle regulators. By controlling the levels of these gene products, zinc-related enzymes can influence processes (e.g., cell proliferation, differentiation, and apoptosis) that are essential for normal tissue development and maintenance in animals [124].

### Zinc-mediated signaling pathways

#### Identification of key signaling pathways

The mitogen-activated protein kinase (MAPK) signaling pathway is among the important pathways regulated by zinc. The MAPK pathway consists of a series of protein kinases, including extracellular-signal-regulated kinases (ERKs), c-Jun N-terminal kinases (JNKs), and p38 MAPKs. These kinases are activated upon exposure to diverse extracellular stimuli, including growth factors, cytokines, and cellular stress signals. Zinc can modulate the activity of the MAPK pathway at multiple levels [125, 126]. For example, zinc can directly interact with certain kinases in the pathway, affecting their phosphorylation and activation status. In some cell types, zinc deficiency has been shown to reduce the activation of ERK, which is involved in cell proliferation and survival. By phosphorylating downstream transcription factors, activated ERK can promote the expression of genes related to cell cycle progression and growth-promoting factors. Thus, the modulation of ERK activation by zinc has a substantial effect on cell growth-related processes. In contrast, JNK and p38 MAPKs are activated primarily by stress signals (e.g., oxidative stress and inflammatory cytokines). Zinc can also regulate the activation of these kinases. In the presence of zinc deficiency, the activation of JNK and p38 MAPKs may be altered, leading to abnormal regulation of stress response genes and potential effects on apoptosis and inflammatory responses. For instance, activated JNK can phosphorylate c-Jun, a transcription factor, and together, they can regulate the expression of genes involved in apoptosis and inflammation [127, 128].

The NF-κB signaling pathway is another key pathway in which zinc plays an important role. NF-κB is a transcription factor that exists in the cytoplasm in an inactive form that is bound to inhibitor of NF-κB (IκB). When cells are stimulated by various factors, including cytokines (e.g., TNF-α), lipopolysaccharides (LPSs) from bacteria, or oxidative stress, the IκB kinase (IKK) complex is activated. This activation leads to the phosphorylation of IκB, which is then ubiquitinated and degraded by the proteasome. As a result, NF-κB is released and translocates into the nucleus, where it binds to specific DNA sequences in the promoter regions of target genes and activates their transcription. Zinc can influence the NF-κB signaling pathway at different steps. It can affect the activation of IKK, either directly or through its impact on upstream signaling molecules [129]. Zinc may also affect the stability and function of NF-κB itself. In immune cells, the activation of NF-κB is crucial for the expression of genes encoding cytokines, chemokines, and other immune-related molecules. For example, in macrophages, the activation of NF-κB in response to LPS stimulation leads to the production of proinflammatory cytokines e.g., TNF-α and interleukin-6 (IL-6), which are important for the initiation and regulation of the immune response [130].

Zinc is involved in other signaling pathways in addition to MAPK and NF-κB pathways. For example, zinc can affect the insulin-like growth factor (IGF) signaling pathway. The IGF pathway is important for growth and development, as it regulates cell growth, proliferation, and differentiation. Zinc may influence the binding of IGF to its receptor or affect downstream signaling events within the pathway. In animals, proper functioning of the IGF signaling pathway is essential for normal growth, and any disruption caused by zinc-related factors can have significant consequences for growth and development [131, 132]. Zinc can also interact with the phosphatidylinositol 3-kinase (PI3K)/Akt signaling pathway, which is involved in cell survival, growth, and metabolism. Through its modulation of this signaling pathway, zinc affects critical cellular processes, including cell cycle progression, apoptosis, and the expression of metabolism-associated genes. For example, the activation of Akt by phosphorylation can lead to the inhibition of apoptosis-promoting factors and the activation of factors involved in cell growth and metabolism-related processes [133].

#### Molecular events in signaling pathways

In the inactive state, NF-κB resides in the cytoplasm and is bound to IκB in a complex. Following extracellular stimulation—for instance, the binding of TNF-α to its cell-surface receptor—a cascade of intracellular signaling events is initiated. Initially, the TNF-α receptor recruits several adaptor proteins, including TNF-receptor-associated factor 2 (TRAF2) and receptor-interacting protein 1 (RIP1). These adaptor proteins form a signaling complex that activates TGF-β–activated kinase 1 (TAK1), which is an upstream kinase in the NF-κB signaling cascade [134].

Zinc can influence this early stage of the signaling process. Zinc may affect the conformation or activity of adaptor proteins. For example, zinc ions may be able to bind to specific cysteine-rich regions in TRAF2, which contain zinc-binding motifs. This binding may stabilize the structure of TRAF2, increasing its ability to interact with other components of the signaling complex and facilitating the activation of TAK1. Under zinc-deficient conditions, the lack of zinc may lead to an unstable conformation of TRAF2, reducing its interaction with other proteins and ultimately reducing the activation of the downstream signaling cascade. Once TAK1 is activated, it phosphorylates and activates the IKK complex. The IKK complex consists of two catalytic subunits, namely, IKKα and IKKβ, and a regulatory subunit, NF-κB essential modulator (NEMO) [135]. Zinc also plays a role in the activation of the IKK complex. Some studies suggest that zinc may directly interact with IKKβ, promoting its phosphorylation and activation. Zinc may also influence the association of the IKK complex components. In the absence of sufficient zinc, the proper assembly and activation of the IKK complex may be disrupted [134].

After the activation of the IKK complex, IKKβ phosphorylates IκB at specific serine residues. This phosphorylation targets IκB for ubiquitination by an E3 ubiquitin ligase complex. Ubiquitinated IκB is then recognized and degraded by the 26S proteasome [136]. Zinc can affect the ubiquitination and degradation process. Zinc finger proteins, which are abundant in cells, may be involved in this process. For example, some zinc finger-containing E3 ubiquitin ligases may be regulated by zinc levels. In a zinc-sufficient environment, these E3 ligases can efficiently ubiquitinate IκB, leading to its degradation and the release of NF-κB [137].

**Fig. 6:**
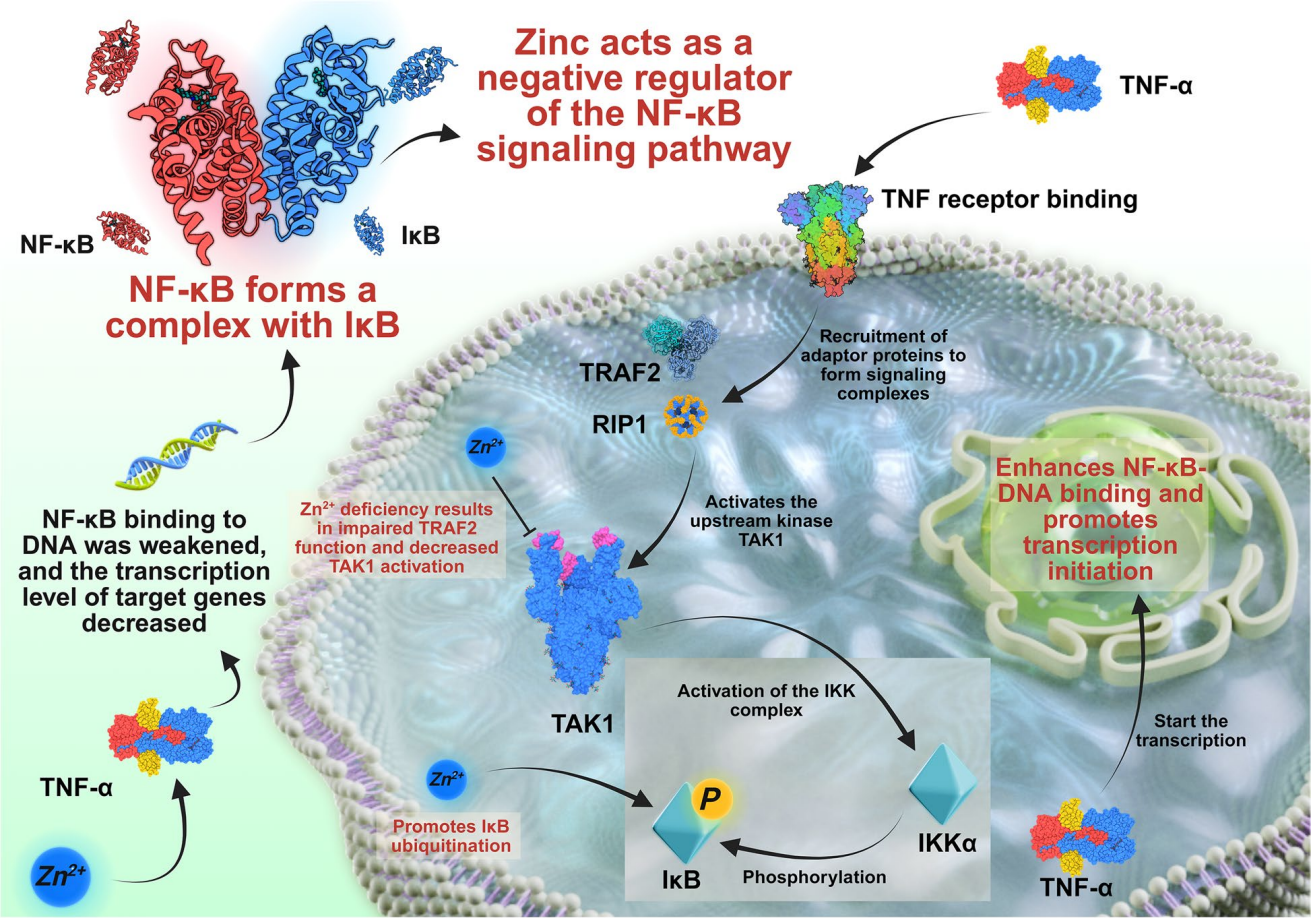
Effects of zinc on the NF-κB signaling pathway

Once NF-κB is released from the IκB complex, it translocates into the nucleus. In the nucleus, NF-κB binds to specific DNA sequences, known as κB sites, in the promoter regions of target genes. Zinc can influence the DNA-binding ability of NF-κB. NF-κB subunits contain Rel homology domains (RHDs) that are involved in DNA binding. Zinc may interact with RHDs, either directly or through other associated proteins, to stabilize the binding of NF-κB to DNA. This interaction can increase the recruitment of RNA polymerase II and other transcription factors to promoter regions, thereby promoting the transcription of target genes. In zinc-deficient cells, the binding of NF-κB to DNA may be impaired, resulting in reduced transcription of genes involved in immune responses, inflammation, and cell survival [138] (Fig. 6).

Overall, the regulation of the NF-κB signaling pathway by zinc involves a complex series of molecular events spanning from the activation of upstream signaling molecules to the regulation of gene expression in the nucleus.

### Zinc-responsive transcription factors and gene regulation

#### Transcription factors sensitive to zinc levels

Transcription factors play a crucial role in regulating gene expression in response to various signals, and some of them are highly sensitive to zinc levels. One of the most well-studied zinc-responsive transcription factors is MTF-1. Its domains are essential for its structure and function. When the intracellular zinc concentration changes, MTF-1 can sense these fluctuations. In the presence of elevated zinc levels, zinc ions bind to the zinc-binding domains of MTF-1. This binding induces a conformational change in MTF-1, enabling it to translocate from the cytoplasm to the nucleus. Once in the nucleus, MTF-1 binds to specific DNA sequences called MREs located in the promoter regions of target genes. MTF-1 is involved primarily in maintaining zinc homeostasis in cells. One of its main target genes is the *MT* gene. When MTF-1 binds to the MREs in the *MT* gene promoter, the transcription of the *MT* gene is activated. As a result, more MT protein is synthesized. The newly synthesized MT can then bind to excess zinc ions in the cell, reducing the free zinc ion concentration and maintaining the zinc balance [139, 140]. This process is crucial for preventing zinc toxicity, as excessive amounts of free zinc ions can be harmful to cells.

In addition to regulating the *MT* gene, MTF-1 regulates the expression of other genes involved in zinc transport and metabolism. For example, it can influence the expression of genes encoding zinc transporters (e.g., *ZnT1* and *ZnT2*). By regulating the expression of these transporters, MTF-1 can control the influx and efflux of zinc across the cell membrane and within the cell, further contributing to the maintenance of zinc homeostasis [141, 142].

Another zinc-sensitive transcription factor is specificity protein 1 (Sp1). Sp1 contains zinc finger domains and is involved in the regulation of a wide range of genes related to cell growth, differentiation, and metabolism. In the context of zinc regulation, Sp1 can be affected by changes in zinc levels. Zinc deficiency can lead to a decrease in the DNA-binding ability of Sp1. Since Sp1 binds to specific GC-rich sequences in the promoter regions of its target genes to activate their transcription, a reduction in its DNA-binding ability due to zinc deficiency can result in the downregulation of these target genes [143]. Some of the target genes regulated by Sp1 are involved in cell cycle progression. For example, the expression of genes encoding cyclins and cyclin-dependent kinases (CDKs), which are crucial for the regulation of the cell cycle, can be affected by Sp1. Under zinc-deficient conditions, the downregulation of these genes mediated by the impaired function of Sp1 can lead to cell cycle arrest or abnormal cell cycle progression, ultimately affecting cell growth and proliferation [144, 145].

#### Mechanisms of gene regulation

Zinc modulates gene expression through zinc-sensing transcription factors (e.g., MTF-1 and Sp1), which detect changes in intracellular zinc levels and orchestrate transcriptional programs to maintain cellular homeostasis [139].

Upon zinc binding, MTF-1 undergoes a conformational change that facilitates its nuclear translocation and binding to MREs in the promoter regions of target genes [146]. This recruitment initiates the assembly of the preinitiation complex, whose components include RNA polymerase II and general transcription factors (e.g., TFIID, TFIIB, and TFIIH), which collectively facilitate transcription initiation [146, 147]. Zinc finger proteins achieve precise transcriptional regulation by recruiting specific coregulators. On one hand, coactivators like p300/CBP enhance transcription by acetylating histones, which relaxes chromatin. On the other hand, corepressors suppress gene expression by recruiting histone deacetylases (HDACs) to deacetylate histones, leading to chromatin compaction [148, 149]. Sp1 is another zinc-sensitive transcription factor that regulates genes related to cell growth and differentiation through GC-rich promoter elements. Zinc deficiency impairs the DNA-binding activity of the transcription factor Sp1, leading to reduced expression of essential cell cycle regulators, e.g., cyclins and CDKS, ultimately causing cell cycle arrest. Additionally, zinc participates in posttranscriptional control by modulating the activity of RNA-binding proteins with zinc finger domains, although the full scope of these mechanisms remains to be clarified in vivo [144, 145].

These precise, zinc-dependent regulatory cascades highlight the key role of zinc in coordinating gene expression in response to physiological needs, ensuring the proper functioning of cellular activities and systemic homeostasis in animals.

## Zinc deficiency and excess in animals

### Zinc deficiency

#### Causes of zinc deficiency

Zinc deficiency in animals is a multifactorial disease, classified as primary and secondary, stemming from an imbalance between dietary supply and physiological demand, often exacerbated by absorption disorders. The main causes include the intake of feed sourced from zinc-deficient soils; the dietary presence of antinutrients (e.g., phytic acid, iron, copper and high calcium), which form insoluble complexes or compete for absorption pathways; and insufficient zinc intake during periods of high need (e.g., growth, pregnancy, and lactation) [10, 23, 25]. Moreover, pathological conditions, e.g., diarrhea that damage intestinal integrity are important contributing factors, making zinc deficiency typically the result of inadequate intake, reduced bioavailability, and increased physiological burden [30].

#### Manifestations and effects of zinc deficiency

Zinc plays a vital role in many enzymatic, structural, and regulatory processes, and zinc deficiency can lead to a series of functional disorders. Growth retardation and skeletal abnormalities arise from disrupted nucleic acid metabolism and impaired osteoblast activity [5, 6]. The atrophy of immune organs and diminished lymphocyte proliferation underscore a compromised immune competence directly linked to the malfunction of zinc finger proteins in cytokine gene regulation [11]. In the context of reproduction, the observed impaired spermatogenesis, hormonal imbalances, and reduced litter sizes are direct consequences of aberrant cell division and gene expression in reproductive tissues [85, 97]. Similarly, dermatitis and poor coat quality result from dysregulated epithelial cell proliferation [45, 46]. Thus, the diverse clinical signs are not isolated events but interconnected symptoms of core metabolic disruption, ultimately leading to diminished animal productivity, welfare, and economic return.

### Zinc excess

#### Sources of and reasons for zinc excess

The main sources of excess zinc in animals are high-zinc diets and environmental zinc pollution. In modern animal husbandry, to pursue high-yield and high-quality production, some farmers may oversupplement animal feed with zinc. For example, in pig farming, owing to the belief that high-zinc diets can promote growth and prevent diarrhea in weaned piglets, the zinc content in some feeds may far exceed the normal requirement. The recommended zinc concentration in pig diets is usually approximately 80–100 mg/kg, but in some cases, the zinc concentration in feed can reach 2000 mg/kg or even higher [150]. This oversupplementation is due mainly to misunderstandings about the role of zinc and the pursuit of short-term economic benefits.

In addition, environmental pollution is an important factor leading to zinc excess in animals. With the development of industry, a large amount of zinc-containing waste is discharged into the environment. Zinc-containing industrial wastewaters and solid wastes can contaminate soil and water sources. Plants grown in zinc-contaminated soil can absorb excessive amounts of zinc, and when animals consume these plants as feed, they may ingest excessive amounts of zinc. For example, in some industrial areas, the zinc content in forage plants is substantially higher than normal because of soil contamination, posing a risk of zinc excess for grazing animals [151]. In addition, the use of zinc-based pesticides and fertilizers in agriculture can increase the zinc content in the soil, further exacerbating the problem of zinc excess in the environment and in animals that rely on these contaminated resources [152].

The use of some zinc-containing drugs or additives in animal husbandry can also lead to zinc excess. For example, in some cases, farmers may use zinc-containing drugs for disease prevention and treatment without following the correct dosage instructions, resulting in excessive zinc intake by animals. Some zinc-based growth-promoting additives, if overused, can also contribute to zinc excess in animals [153].

#### Harmful effects of zinc excess

Excess of zinc can have a series of harmful effects on animals, manifested mainly in its interference with the absorption and utilization of other minerals, its impact on enzyme activity, and the potential risk of promoting the emergence of antibiotic-resistant bacteria [150, 154].

Excessive zinc can interfere with the absorption and utilization of other essential minerals in animals. Zinc has a competitive relationship with copper, iron, and selenium in the process of absorption. When the zinc content in the diet is too high, the absorption of copper can be reduced. High-zinc diets can lead to a decrease in the activity of copper-containing enzymes, including ceruloplasmin, which is involved in iron metabolism. This can further cause iron deficiency anemia in animals [155, 156]. In addition, excessive zinc can inhibit the absorption of selenium, affecting the activity of selenium-containing enzymes, e.g., glutathione peroxidase, which is important for antioxidant defense in the body. For example, in chickens fed high-zinc diets, the contents of copper and iron in the liver and blood are significantly reduced, and the antioxidant capacity of the body is decreased because of the inhibition of selenium absorption [157].

The activity of some enzymes can be affected by excess zinc. Phytase is an enzyme that can hydrolyze phytic acid in feed, increasing the availability of phosphorus and other nutrients. However, high levels of zinc can inhibit the activity of phytase. In the presence of excessive zinc, the catalytic efficiency of phytase is reduced, resulting in less efficient hydrolysis of phytic acid. This not only reduces the utilization rate of phosphorus in the feed but also increases the amount of phosphorus excreted by animals, which can cause environmental pollution [158]. For example, in pig production, if the feed contains excessive amounts of zinc, the activity of phytase in the digestive tract of pigs is inhibited, leading to lower phosphorus utilization and higher phosphorus excretion in feces [159].

Another potential harmful effect of zinc excess is the promotion of the emergence of antibiotic-resistant bacteria. Some studies have shown that high-zinc environments can increase the resistance of bacteria to antibiotics. Zinc can interact with the cell membranes of bacteria, altering their permeability and structure, possibly leading to the upregulation of genes related to antibiotic resistance in bacteria. For example, in the gut microbiota of animals fed high-zinc diets, the proportion of antibiotic-resistant bacteria is greater than that in animals with normal zinc intake. This poses a threat to animal health and the safety of animal-derived products, as well as to human health when these antibiotic-resistant bacteria are transmitted through meat products [160].

In contrast to zinc deficiency, which is often a consequence of inadequate intake or absorption, zinc excess typically arises from deliberate dietary oversupplementation or environmental contamination [151]. While zinc deficiency leads to systemic impairment of biological processes, zinc excess exerts its toxicity primarily through the competitive inhibition of the absorption of other essential minerals (e.g., Cu and Fe) and the disruption of the gut microbial ecology, potentially promoting antibiotic resistance. Both states of imbalance highlight the critical importance of precise zinc management in animal nutrition [70].

## Application and prospects of zinc in animal production

### Application of zinc in animal feed

Dietary supplementation of zinc in animal feed is achieved using two categories of sources: inorganic and organic zinc compounds.

Inorganic zinc sources (e.g., zinc oxide and zinc sulfate) are widely used in animal feed. Zinc oxide is a white hexagonal crystal or powder that is odorless and insoluble in water but soluble in acid and sodium hydroxide solutions. It has a relatively high zinc content, with an elemental zinc content of approximately 72%. In the past, high-dose zinc oxide (2,000–3,000 mg/kg) was often added to the diet of weaned piglets [150]. The addition of this high dose can effectively reduce the diarrhea rate in weaned piglets. The mechanism may be related to its antibacterial properties, as well as its ability to promote the repair of intestinal mucosa. For example, it can increase the expression of tight junction proteins in the intestinal epithelium, reducing intestinal permeability and preventing the invasion of pathogens. However, the prolonged administration of high-dose zinc oxide presents several concerns. Notably, the elevated fecal zinc excretion that contributes to environmental contamination. Furthermore, it may impair the absorption of essential minerals, including iron and copper, in animals [161].

Zinc sulfate exists in a monohydrate (with an elemental zinc content of approximately 35.5%) and a heptahydrate (with an elemental zinc content of approximately 22.3%) form. Owing to its relatively low price and specific bioavailability, zinc sulfate is among the most used zinc sources in animal feed. Studies on poultry production have shown that the addition of zinc sulfate to the diet of laying hens can improve eggshell quality. For instance, when a certain amount of zinc sulfate is added to the diet of laying hens during the late laying period, it can improve the microstructure of the eggshell, making the eggshell more compact and reducing the proportion of broken eggs [162]. In livestock production, zinc sulfate is typically employed to meet the fundamental zinc requirements of animals. It participates in diverse metabolic processes, notably by serving as a cofactor for enzymes involved in protein and carbohydrate metabolism.

Organic zinc sources, including zinc gluconate and zinc methionine, have attracted increasing attention in recent years. Zinc gluconate is a compound formed by the combination of zinc ions and gluconic acid. It has relatively high solubility and stability in water. In animal experiments, adding zinc gluconate to the diet of rats has been shown to improve their growth performance [163]. It is readily absorbed and utilized by animals and contributes to key physiological functions, including the synthesis of proteins and nucleic acids. Compared with some inorganic zinc sources, the relatively mild chemical properties of zinc gluconate also reduce its potential negative impact on the digestive tract of animals [164, 165].

Zinc methionine, an amino acid-chelated form of zinc, has unique advantages. It is formed by the chelation of zinc ions with methionine. This form of zinc is more similar to the form in which zinc exists in the animal body; thus, it has higher bioavailability than other forms. In pig production, the addition of zinc methionine to the diet can increase their feed conversion rate and growth rate. For example, in a study on growing-finishing pigs, the addition of an appropriate amount of zinc methionine increased the average daily gain of the pigs and reduced the feed-to-gain ratio. The reason may be that zinc methionine can be directly absorbed by the small intestine through the amino acid transport system, bypassing some of the complex absorption processes of inorganic zinc. In addition, zinc methionine can improve the immune function of animals. In poultry, the addition of zinc methionine to the diet of broilers can increase the weight of immune organs (e.g., the thymus and spleen) and improve the immune response of the broilers to pathogens, thus reducing the incidence of diseases [166].

### Current research hotspots and future prospects

In the field of zinc in animal nutrition, several research hotspots have emerged in recent years, with significant implications for both the theoretical understanding of this topic and its practical applications in animal production.

The development of novel zinc sources is a key area of research. Traditional inorganic zinc sources have been widely used but also have limitations. For example, high-dose zinc oxide, although effective at preventing diarrhea in weaned piglets, leads to the excretion of large amounts of zinc in feces, causing environmental pollution. In response, researchers are focusing on developing more efficient and environmentally friendly zinc sources. Organic zinc sources, e.g., zinc methionine and zinc gluconate, have shown great potential. Zinc methionine, an amino acid-chelated form of zinc, has higher bioavailability than other forms because it can be directly absorbed by the small intestine through the amino acid transport system. Studies have demonstrated that the addition of zinc methionine to the diet of pigs can increase their feed conversion rate and growth rate and improve their immune function [167, 168]. Moreover, new forms of zinc-based compounds are being explored. Studies exploring the development of zinc-based nanoparticles, which exhibit unique properties, e.g., improved bioavailability and targeted delivery within animals, is ongoing. These novel zinc sources could provide greater nutritional value while reducing the negative environmental impacts associated with traditional zinc sources [169, 170].

Precision nutrition regulation related to zinc is another hot topic. With the development of modern animal nutrition science, there is an increasing emphasis on formulating diets that precisely meet the zinc requirements of different animals at various growth stages. This task necessitates a thorough understanding of the factors influencing zinc metabolism in animals, including the effects of various feed components on zinc bioavailability and the distinct zinc requirements during critical physiological processes and states including growth, reproduction, and immune challenge [169]. For example, the genetic backgrounds of different livestock breeds may influence their zinc utilization efficiency. By studying these genetic differences, it is possible to develop personalized zinc supplementation strategies for different breeds. Moreover, advanced techniques (e.g., metabolomics and proteomics) can provide comprehensive analyses of alterations in metabolic pathways and protein expression profiles in response to zinc supplementation. This information can be used to optimize the zinc supplementation level and timing to achieve more efficient utilization of zinc in animal production [158, 171, 172].

Future research directions in this field are likely to focus on several aspects. More in-depth studies on the molecular mechanisms of zinc-related physiological functions are the primary need. Although substantial progress has been made in understanding the role of zinc in enzyme-mediated reactions, signaling pathways, and gene regulation, many unknowns remain. For example, the exact molecular mechanisms through which zinc-responsive transcription factors interact with other regulatory elements in different cell types during various physiological and pathological processes need to be further explored. Such findings could lead to a more comprehensive understanding of how zinc affects animal health at the molecular level and provide a basis for the development of more targeted zinc supplementation strategies [173].

Secondly, the relationship between zinc and the gut microbiota in animals is expected to be an important research area. The gut microbiota plays a crucial role in animal health, influencing digestion, immunity, and metabolism. Zinc can affect the composition and function of the gut microbiota, and vice versa. Future studies are likely to prioritize clarifying the precise interactions between zinc and the gut microbiota—particularly how zinc supplementation regulates the composition and function of beneficial intestinal bacteria and how zinc-induced microbial disruptions influence overall animal health [160]. This knowledge could be used to develop probiotic–zinc combination strategies to improve animal gut health and overall performance.

Finally, considering the environmental impact of zinc use in animal production, research on sustainable zinc management practices is anticipated to be essential. Topics include reducing zinc excretion in animal waste through more efficient zinc utilization strategies and exploring ways to recycle and reuse zinc in the animal production cycle. For example, developing feed processing technologies that can improve the bioavailability of zinc in feed, reducing the need for high-dose zinc supplementation, and exploring methods to recover zinc from animal manure for potential reuse in agriculture or other industries are potential research foci [174, 175]. Such sustainable practices are crucial for the long-term development of the animal husbandry industry, ensuring both animal health and environmental protection.

## Summary

Zinc is an essential trace element for physiological functions in animals and is crucial for maintaining normal physiological functions. It supports growth and skeletal development, regulates immune function through zinc finger proteins, and affects immune organ integrity, lymphocyte proliferation, and cytokine expression. Zinc also affects the reproductive system; zinc deficiency can impair sperm production, delay puberty in males, and reduce the litter size and embryo survival rate in females.

At the molecular level, zinc precisely maintains homeostasis by regulating the activity of enzymes (e.g., SOD), signaling pathways (e.g., MAPK and NF-κB pathways), and transcription factors, e.g., MTF-1 and Sp1. Either excessive or deficient zinc intake can disrupt mineral balance, interfere with enzyme functions, and disturb the gut microbiome, leading to metabolic and health issues.

In animal production, both inorganic zinc (e.g., zinc oxide and zinc sulfate) and organic zinc (e.g., zinc methionine and zinc gluconate) forms contribute to growth promotion and immune enhancement. However, their excessive use poses environmental risks. Future research should prioritize the development of novel zinc sources (e.g., nano zinc), the implementation of precision nutrition strategies using omics-based dynamic models, and the elucidation of mechanisms governing zinc–gut microbiota interactions in immune and metabolic regulation. These approaches will facilitate the development of targeted strategies to improve animal health and promote sustainable livestock farming.

## Data Availability

Not applicable.

## Abbreviations

Akt: Protein kinase B
CDKs: Cyclin-dependent kinases
ERK: Extracellular-signal-regulated kinases
IκB: Inhibitor of NF-κB
IKK: IκB kinase
IGF: Insulin-like growth factor
IL-1: Interleukin-1
IL-2: Interleukin-2
IL-6: Interleukin-6
JNK: C-Jun N-terminal kinases
LPS: Lipopolysaccharides
MAPK: Mitogen-activated protein kinase
MREs: Metal-response elements
MT: Metallothionein
MTF-1: Metal-regulatory transcription factor 1
NF-κB: Nuclear factor kappa B
NK cells: Natural killer cells
PI3K: Phosphatidylinositol 3-Kinase
ROS: Reactive oxygen species
SOD: Superoxide dismutase
Sp1: Specificity protein 1
TNF-α: Tumor necrosis factor-alpha
TRAF2: TNF receptor-associated factor 2
ZnT1: Zinc transporter 1
ZnT2: Zinc transporter 2
ZnT3: Zinc transporter 3
ZnT4: Zinc transporter 4
